# Detrimental role of the EP1 prostanoid receptor in blood-brain barrier damage following experimental ischemic stroke

**DOI:** 10.1038/srep17956

**Published:** 2015-12-09

**Authors:** Jan C. Frankowski, Kelly M. DeMars, Abdullah S. Ahmad, Kimberly E. Hawkins, Changjun Yang, Jenna L. Leclerc, Sylvain Doré, Eduardo Candelario-Jalil

**Affiliations:** 1 Department of Neuroscience, McKnight Brain Institute, University of Florida, Gainesville, FL 32610, USA; 2 Department of Anesthesiology, University of Florida, Gainesville, FL 32610, USA

## Abstract

Cyclooxygenase-2 (COX-2) is activated in response to ischemia and significantly contributes to the neuroinflammatory process. Accumulation of COX-2-derived prostaglandin E_2_ (PGE_2_) parallels the substantial increase in stroke-mediated blood-brain barrier (BBB) breakdown. Disruption of the BBB is a serious consequence of ischemic stroke, and is mainly mediated by matrix metalloproteinases (MMPs). This study aimed to investigate the role of PGE_2_ EP1 receptor in neurovascular injury in stroke. We hypothesized that pharmacological blockade or genetic deletion of EP1 protects against BBB damage and hemorrhagic transformation by decreasing the levels and activity of MMP-3 and MMP-9. We found that post-ischemic treatment with the EP1 antagonist, SC-51089, or EP1 genetic deletion results in a significant reduction in BBB disruption and reduced hemorrhagic transformation in an experimental model of transient focal cerebral ischemia. These neurovascular protective effects of EP1 inactivation are associated with a significant reduction in MMP-9/-3, less peripheral neutrophil infiltration, and a preservation of tight junction proteins (ZO-1 and occludin) composing the BBB. Our study identifies the EP1 signaling pathway as an important link between neuroinflammation and MMP-mediated BBB breakdown in ischemic stroke. Targeting the EP1 receptor could represent a novel approach to diminish the devastating consequences of stroke-induced neurovascular damage.

Ischemic stroke is characterized by the occlusion of an artery supplying the brain, resulting in neuronal death within minutes in the infarct core. Surrounding the infarct core is the penumbra, an area of tissue that is susceptible to infarction, but is potentially salvageable. Injury to the brain expands from the infarct core to the penumbra and involves numerous mechanisms including ionic imbalances, oxidative stress, neuroinflammation, immune cell infiltration, and disruption of the blood-brain barrier (BBB)^1^,^2^. The BBB is comprised of endothelial cells, tight-junction proteins (TJPs), extracellular matrix proteins, astrocytes, pericytes, and perivascular microglia, which together form a highly selective barrier between circulating blood and the brain^3^,^4^.

Disruption of the BBB is a serious consequence of ischemic stroke, and is mainly mediated by matrix metalloproteinases (MMPs), a family of enzymes that degrade the TJPs and extracellular matrix^5^,^6^,^7^,^8^. A large body of preclinical and clinical evidence indicates that MMP-3 and MMP-9 are key effectors of neurovascular damage, vasogenic edema, and hemorrhagic transformation in ischemic stroke^7^,^9^,^10^,^11^,^12^. Pharmacological inhibition or genetic deletion of MMP-3 and MMP-9 is beneficial in animal models of ischemic brain injury^8^,^11^,^12^,^13^,^14^. Several studies indicate that damage to the BBB is a significant contributor to progressive neuronal death in the penumbral region after stroke^8^,^15^. Therefore, understanding mechanisms responsible for neurovascular damage is instrumental for developing an effective therapy in human ischemic stroke.

Neuroinflammatory processes significantly contribute to the pathophysiology of ischemic stroke. Cyclooxygenase-2 (COX-2) is activated in response to ischemic brain injury and catalyzes the production of lipid mediators, many of which are pro-inflammatory and detrimental to the ischemic tissue^16^,^17^,^18^,^19^. COX-2 inhibition reduces BBB permeability and MMP activity in animal models of ischemic stroke and neuroinflammation^20^,^21^. Prostaglandin E_2_ (PGE_2_) is a major product of increased COX-2 activity during inflammatory conditions and cerebral ischemia^18^,^20^. Accumulation of COX-2-derived PGE_2_ in the ischemic brain parallels the substantial increase in BBB breakdown and neutrophil infiltration^20^. There is *in vivo* evidence indicating that intracerebral injection of PGE_2_ leads to a significant increase in BBB permeability^22^.

PGE_2_ exerts its actions through four E prostanoid (EP) receptors, termed EP1 through EP4^23^. Activation of the EP1 receptor is an important mechanism associated with the detrimental effects of COX-2-derived PGE_2_ in experimental ischemic stroke^24^,^25^,^26^,^27^. Over the past decade, numerous studies have demonstrated that pharmacological inhibition or genetic inactivation of the EP1 receptor confers neuroprotection, both in *in vitro* and *in vivo* models of ischemic injury by countering excitotoxicity^24^,^26^,^27^ and apoptotic signaling^28^,^29^,^30^. However, virtually nothing is known of the role of EP1 in the neuroinflammatory events resulting in BBB damage in stroke. We hypothesized that pharmacological inhibition or genetic deletion of EP1 protects against BBB damage and hemorrhagic transformation by decreasing the levels and activity of MMP-9/-3. Utilizing the ischemic stroke model of middle cerebral artery occlusion (MCAO), we tested the impact of EP1 pharmacological blockade or EP1 genetic deletion on neurovascular injury after ischemia. We found that post-ischemic treatment with the EP1 receptor antagonist, SC-51089, or EP1 genetic deletion results in a significant reduction in BBB disruption and reduced hemorrhagic transformation following transient focal cerebral ischemia. These BBB protective effects of EP1 inactivation are associated with a significant reduction in MMP-9/-3, less peripheral neutrophil infiltration, and a preservation of tight junction proteins composing the neurovascular unit. Our study identifies the EP1 signaling pathway as an important link between neuroinflammation and MMP-mediated BBB breakdown in ischemic stroke. Targeting the EP1 receptor could represent a novel approach to diminish the devastating consequences of stroke-induced neurovascular damage.

## Results

### EP1 is upregulated following ischemic stroke, expressed on neurons, and endothelial cells

**Figure 1:**
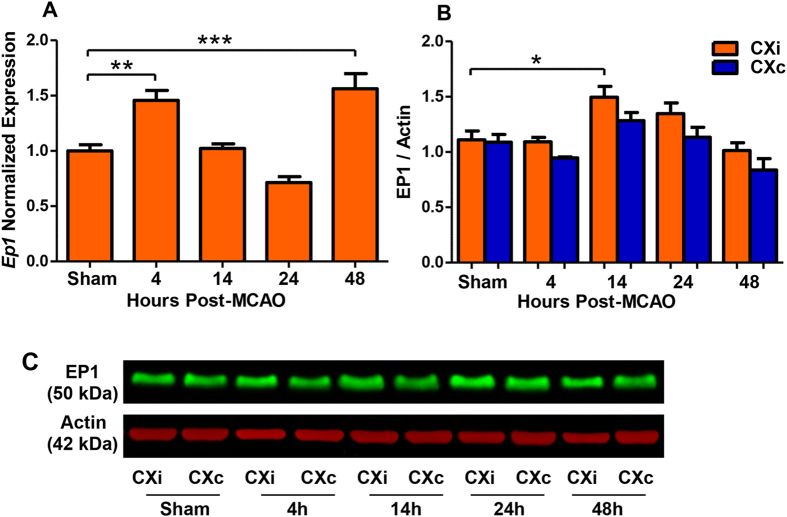
Time course of EP1 expression after cerebral ischemia in rats. *EP1* mRNA expression in the ischemic cortex and EP1 protein levels in the ischemic and contralateral hemispheres were measured in sham-operated and 4, 14, 24, and 48 h after the onset of occlusion. (**A**) *EP1* mRNA is increased at 4 h (P < 0.01, one-way ANOVA) and 48 h after occlusion (P < 0.001, one-way ANOVA) compared to the sham. (**B**) EP1 protein levels are elevated in the ischemic cortex 14 h after occlusion compared to the sham (P < 0.05, two-tailed unpaired t-test). (**C**) A representative immunoblot of EP1 expression in the cortex over time in response to ischemia. Sham, N = 5; 4 h, N = 5; 14 h, N = 6; 24 h, N = 8; 48 h, N = 7. CXi = Cortex ipsilateral to stroke; CXc = Cortex contralateral to stroke.

It is unknown whether the EP1 receptor is differentially regulated in response to ischemia. An increase in expression of EP1 after ischemia could potentiate the receptor’s detrimental effects associated with the neuroinflammatory response to stroke. A time course of ischemic injury was constructed consisting of sham-operated and ischemic rats sacrificed at 4, 14, 24, and 48 hours following MCAO. *EP1* mRNA expression was increased in the ipsilateral cerebral cortex at 4 h (P < 0.01) and 48 h (P < 0.001) following ischemia compared to the expression levels in the ipsilateral cortex of the sham group (Fig. 1A). Next, we sought to assess whether an increase in *EP1* mRNA expression is followed by increased EP1 protein levels. Immunoblotting analyses showed a significant increase in EP1 protein levels at 14 h following ischemia in the ipsilateral cortex compared to the sham group (P < 0.05, Fig. 1B,C).

**Figure 2:**
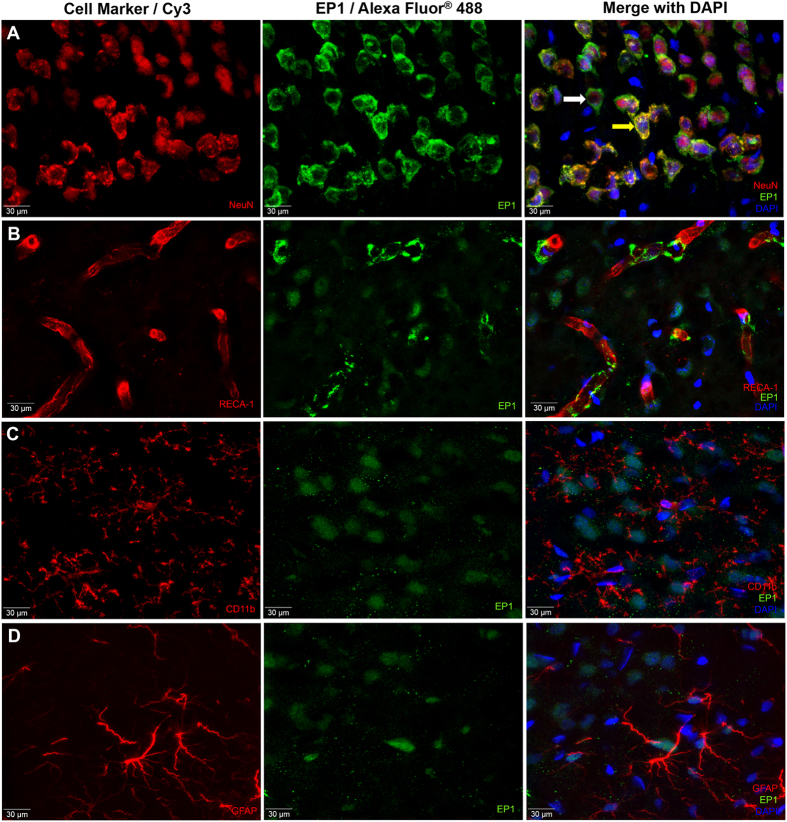
EP1 is expressed on rat neurons and endothelial cells, but not astrocytes or microglia. Free-floating double immunohistochemistry was performed using antibodies against EP1 and cell markers for neurons (NeuN), endothelial cells (RECA-1), astrocytes (GFAP), and microglia (CD11b) in sham-operated rats. All images were taken from cortical tissue. (**A**) NeuN (red) and EP1 (green) double-labeling shows both nuclear (yellow arrow) and extranuclear staining (white arrow) suggesting that EP1 protein may be expressed on the membrane and the nuclear envelope in neurons. (**B**) RECA-1 (red) and EP1 (green) double-labeling demonstrates that EP1 is expressed on endothelial cells. (**C**) CD11b (red) and EP1 (green) double-labeling shows apparent staining on neurons, but not on microglia. (**D**) GFAP (red) and EP1 (green) double labeling demonstrates apparent EP1 staining on neurons, but not on astrocytic processes.

Previous reports on the role of the EP1 in ischemia have focused mainly on neuronal EP1 signaling. Additional cell types may contribute to EP1 signaling after ischemic stroke. To determine what cell types express EP1, we performed double immunofluorescence staining with specific cell markers for neurons, endothelial cells, astrocytes, and microglia. In sham-operated rats, EP1 was found on neurons and endothelial cells (Fig. 2A,B). EP1 staining was not observed on microglia or astrocytes (Fig. 2C,D). A similar EP1 cellular localization was found in the ischemic rat brain at 24 and 48 h following transient focal ischemic stroke (data not shown).

### Delayed treatment with SC-51089, an EP1 antagonist, reduces cortical infarct volume

**Figure 3:**
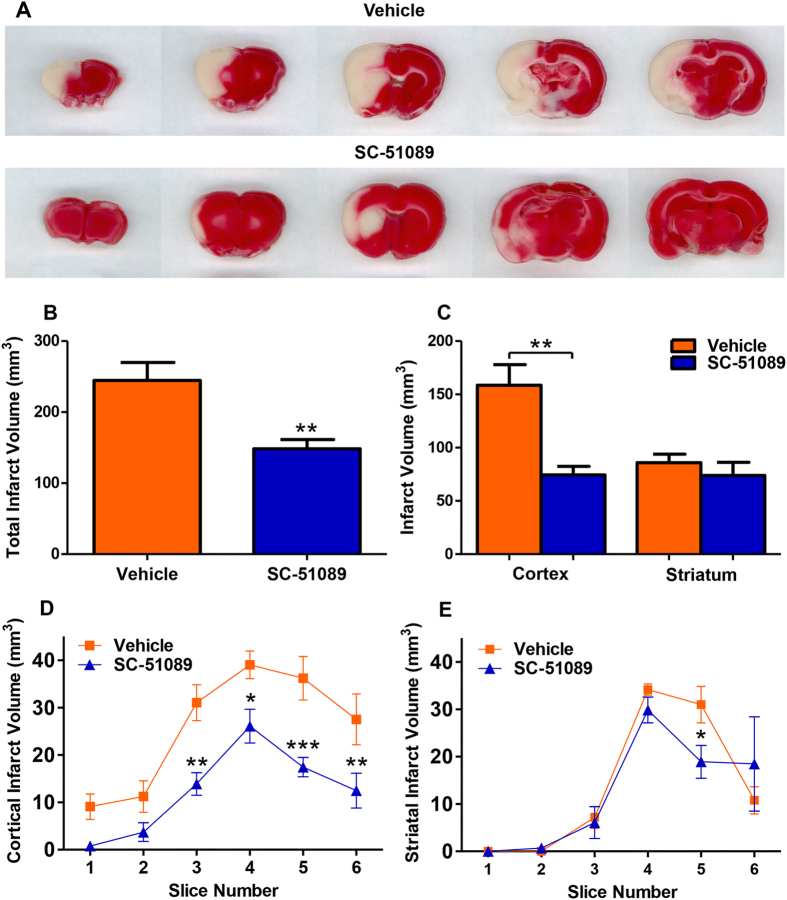
EP1 antagonist administration reduced cortical infarct volume in rats. Rats were subjected to 90 minutes of middle cerebral artery occlusion and euthanized after 48 h of reperfusion. Infarct volume was determined by 2,3,5-triphenyl-2H-tetrazolium chloride staining in both the cortex and striatum. (**A**) Coronal sections of vehicle-treated and SC-51089-treated rats corresponding to slice 1, 2, 3, 5, and 6. The volume of slice 4 is determined by inverting the third section. Rats with cortical infarct volumes closest to the group average are presented. (**B**) SC-51089 administration reduces total infarct volume in rats (148.3 ± 13.2 mm^3^) compared to the vehicle (244.5 ± 25.4 mm^3^, P < 0.01, two-tailed unpaired t-test). (**C**) Analysis of cortical and striatal tissue separately shows nearly identical striatal infarct volumes between SC-51089-treated (73.9 ± 12.3 mm^3^) and vehicle-treated (85.9 ± 7.9) rats (P = 0.43, two-tailed unpaired t-test). Cortical infarct volume was dramatically reduced in SC-51089-treated (83.07 ± 7.721 mm^3^) compared to the vehicle (154.0 ± 18.06 mm^3^). (**D**) Analysis of cortical infarct volume per-2mm slice shows that decreases in cortical infarct volume are seen throughout the cortex (P > 0.05, P > 0.05, P < 0.01, P < 0.05, P < 0.001, P < 0.05, two-way ANOVA w/Bonferroni post-tests). (**E**) Analysis of striatal infarct volume per-2mm section shows that infarct volumes are nearly identical between groups (P > 0.05, P > 0.05, P > 0.05, P > 0.05, P < 0.05, P > 0.05, two- way ANOVA w/Bonferroni post-tests). Vehicle N = 10, SC-51089 N = 11.

There is evidence that SC-51089 reduces infarct size in *mouse* stroke models when given after the onset of MCAO^24^,^31^. However, the ability of SC-51089 to reduce infarct volume in the rat intraluminal stroke model has not been previously investigated. Therefore, infarct volume in the cortex and striatum was determined after 48 h of reperfusion in rats that received SC-51089 or the vehicle starting at 4.5 h after the onset of stroke, which is a clinically relevant delayed treatment schedule. Representative coronal sections from both groups are shown in Fig. 3A. SC-51089 administration reduced total infarct volume by 37% (Fig. 3B, *P* < 0.01). The reduction in infarct volume was localized to the cortex; SC-51089 treatment reduced cortical infarct volume by 52% (Fig. 3C, *P* < 0.01) and no change in total striatal infarct volume was observed (Fig. 3C, *P* = 0.43). We also examined infarct volume per 2-mm slice, slice 1 being the most rostral. Reductions in cortical infarct size were observed in slices 3–6 in the SC-51089-treated group (*P* < 0.01, *P* < 0.05, *P* < 0.001, *P* < 0.05, respectively Fig. 3D). Striatal infarct volume was reduced in slice 5 in the SC-51089-treated group (*P* < 0.05, Fig. 3E). These data indicate that blockade of EP1 with SC-51089 after ischemic stroke protects the outlying cortex and not the striatum.

In this MCAO model, the striatum is damaged immediately by excitotoxicity and oxidative stress, and the cerebral cortex surrounding the striatal infarct core succumbs to cell death over time due to secondary mechanisms, including BBB disruption and neuroinflammation. Since no change in infarct volume was observed in the striatum, and we are interested in protecting the cortex from secondary damage, we performed the rest of our experiments using cortical tissue samples.

### Post-ischemic administration of SC-51089 reduces stroke-induced BBB disruption in rats

**Figure 4:**
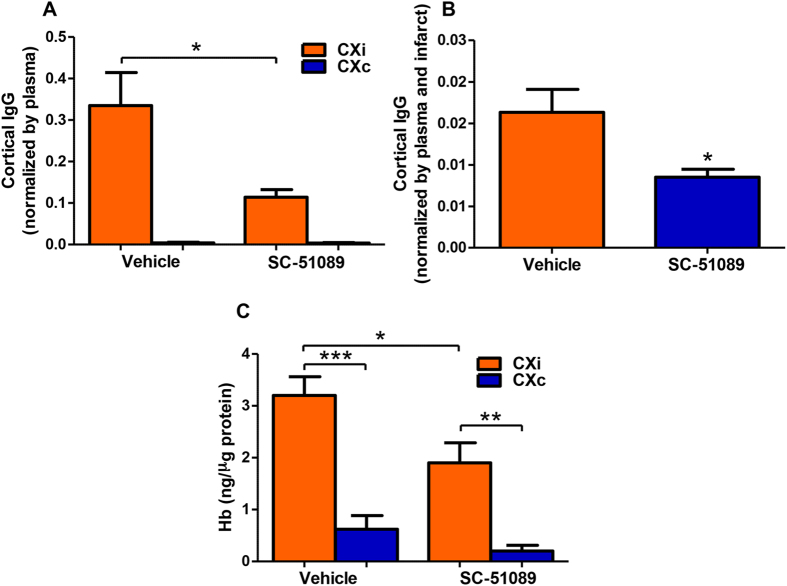
EP1 antagonist administration reduces blood-brain barrier permeability and hemorrhagic transformation. Immunoglobulin G and hemoglobin levels are extremely low in the healthy brain, therefore were used as indicators of the degree of blood-brain barrier permeability and hemorrhagic transformation, respectively. (**A**) IgG concentrations in the cortex were divided by plasma concentrations to yield a corrected measure of blood-brain barrier permeability. Corrected IgG levels are reduced in the ischemic cortex of SC-51089-treated rats compared to the vehicle (P < 0.05). (**B**) Cortical IgG concentration were divided by the plasma concentration and infarct volume of the section used for molecular analyses gives an approximate measure of IgG per cubic volume of infarcted tissue. IgG/unit of infarcted tissue is reduced in SC-51089-treated animals compared to the vehicle (P < 0.01, unpaired two-tailed t-test). (**C**) Average hemoglobin levels were significantly reduced in SC-51089-treated rats (1.90 ± 0.39 ng/μg) compared to the vehicle-treated group (3.20 ± 0.36 ng/μg) (P = 0.0243, two-tailed unpaired t-test). Vehicle N = 10, SC-51089 N = 11. CXi = Cortex ipsilateral to stroke; CXc = Cortex contralateral to stroke.

Disruption of the BBB leads to the uncontrolled influx of blood components into the brain, causing fluid edema and the accumulation of neurotoxic substances, which contribute to the progression of injury after ischemic stroke. To quantify the degree of BBB permeability at 48 h following stroke, levels of immunoglobulin G (IgG) were measured in the ipsilateral and contralateral cortices of SC-51089- and vehicle-treated rats. In both SC-51089 and vehicle groups, IgG concentrations were elevated in the cortex ipsilateral to the stroke (CXi) compared to the contralateral hemisphere (CXc) (Supplemental Fig. 1A, *P* < 0.0001) and IgG levels were greatly reduced in the ischemic hemisphere of SC-51089-treated rats compared to the vehicle (Supplemental Fig. 1A, *P* < 0.001). Using soluble plasma proteins as a marker of BBB permeability assumes protein levels are consistent between animals; to confirm this, IgG levels in plasma were quantified. No changes in IgG levels were observed in the plasma of treated rats compared to the vehicle (Supplemental Fig. 1B, *P* = 0.31). Cortical IgG levels were divided by the corresponding animal’s plasma IgG concentration to yield a corrected measure of BBB permeability; SC-51089 treatment maintained lower IgG levels in the ischemic cortex after correction (Fig. 4A, P < 0.05).Finally, to determine if the reduction of IgG in the cerebral cortex was a function of proportionately reduced stroke volume or if reduction of IgG levels was an effect of treatment with SC-51089, we divided cortical IgG levels by the plasma IgG concentration and by the corresponding infarct volume. Normalized IgG levels were reduced in SC-51089-treated rats compared to the vehicle group, indicating the reductions in IgG levels in the brain are an effect of the treatment (Fig. 4B, *P* < 0.05).

Hemorrhagic transformation occurs in ischemic stroke due to compromised BBB integrity, allowing unregulated influx of blood into the brain^32^. Hemoglobin (Hb) levels were quantified using ELISA in the cortices of SC-51089 and vehicle-treated rats to measure the degree of hemorrhagic transformation. Ipsilateral Hb content was dramatically increased in response to 90 min of MCAO and 48 h of reperfusion in both treatment groups (Fig. 4C, P < 0.01). SC-51089 administration significantly reduced hemoglobin levels in the ischemic cortex compared to the vehicle treated group (Fig. 4C, *P* < 0.05). Altogether, these data suggest that pharmacological blockade of EP1 reduces the degree of BBB permeability and hemorrhagic transformation following ischemic stroke.

### SC-51089 reduces MMP-9 and MMP-3 protein levels and activity in rats

**Figure 5:**
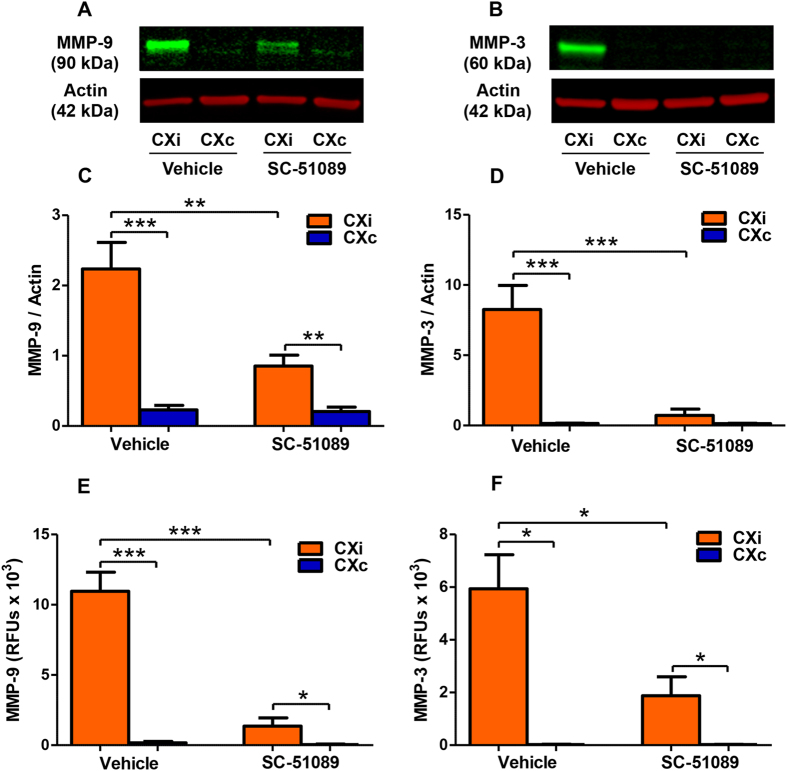
EP1 antagonist administration reduces MMP-9 and MMP-3 protein levels and activity in the ischemic rat cortex. Matrix metalloproteinase-9 and -3 protein levels and enzymatic activity were quantified in the cortices of SC-51089- and vehicle-treated rats.(**A**) A representative immunoblot for MMP-9 in the cortex. (**B**) A representative immunoblot for MMP-3 in cerebral cortex of vehicle- and SC-51089-treated groups. (**C**) Densitometric analysis shows increased MMP-9 levels in the ischemic hemisphere of both groups (P < 0.001, P < 0.01, unpaired two-tailed t-test) compared to the contralateral hemisphere. MMP-9 levels were reduced in the ischemic cortex of SC-51089-treated rats compared to the vehicle group (P < 0.01, unpaired two-tailed t-test). (**D**) Densitometric analysis shows increased MMP-3 levels in the ischemic cortex of vehicle-treated rats (P < 0.001), but not in SC-51089-treated rats (P = 0.20). Reduced MMP-3 levels were measured in the ischemic cortex of SC-51089-treated rats compared to the vehicle group (P < 0.001, unpaired two-tailed t-test). (**E**) A FRET peptide activity assay shows increased MMP-9 activity in the ischemic cortex compared to the contralateral hemisphere in both SC-51089- and vehicle-treated groups (P < 0.001, P < 0.05). Reduced MMP-9 activity was measured in the ischemic cortex of SC-51089-treated rats compared to the vehicle group (P < 0.0001, unpaired two-tailed t-test). (**F**) A FRET peptide activity assay shows increased MMP-3 activity in the ischemic cortex compared to the contralateral hemisphere in both groups (P < 0.05). Reduced MMP-3 activity was measured in the ischemic cortex of SC-51089-treated rats compared to the vehicle group (P < 0.05, unpaired two-tailed t-test). Vehicle N = 10, SC-51089 N = 11. CXi = Cortex ipsilateral to stroke, CXc = Cortex contralateral to stroke, RFUs = Relative Fluorescent Units.

MMP-9 and MMP-3 are two MMPs involved in acute injury in ischemic stroke. MMP-3 cleaves the inhibitory domain of pro-MMP-9, and both active proteases degrade the BBB and extracellular matrix. MMP-9/-3 protein levels in the ischemic brain were measured by immunoblotting (Fig. 5A,B). As expected, MMP-9 and MMP-3 levels were elevated in the ischemic cortex compared to the contralateral hemisphere in vehicle-treated rats (Fig. 5C,D, *P* < 0.001) and nearly undetectable in the contralateral cortex in both SC-51089 and vehicle groups. SC-51089 administration reduced the levels of both MMP-9 (Fig. 5C, *P* < 0.01) and MMP-3 (Fig. 5D, *P* < 0.001) in the ipsilateral cortex compared to the vehicle group. In order to measure endogenously active MMP levels, a fluorometric immunocapture assay was used to quantify the activity of MMP-9 and MMP-3 in the cortices of vehicle- and SC-51089-treated rats. SC-51089 administration significantly reduced the activities of both MMP-9 (Fig. 5E, *P* < 0.0001) and MMP-3 (Fig. 5F, *P* < 0.05) in the ischemic cortex compared to the vehicle.

### SC-51089 reduces neutrophil infiltration in rats

**Figure 6:**
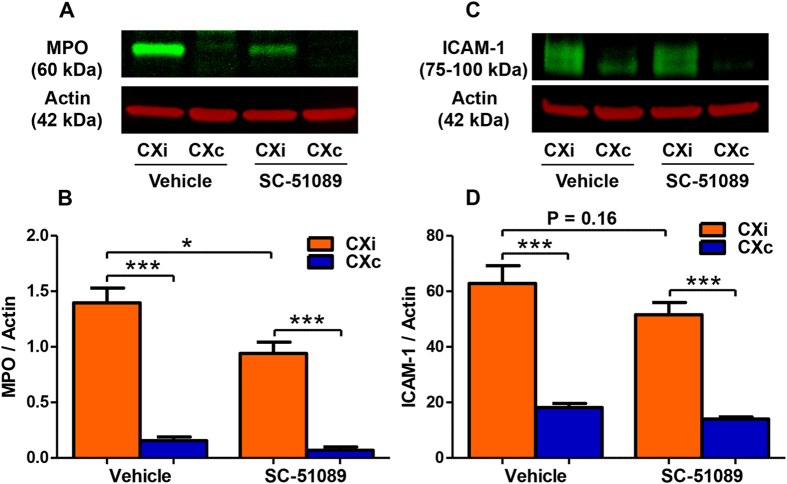
Effects of EP1 antagonist on Myeloperoxidase (MPO) and Intercellular Adhesion Molecule 1 (ICAM-1) levels in the ischemic rat cortex. Myeloperoxidase levels were measured as an indicator of neutrophil infiltration in the ischemic cortex. ICAM-1 levels were measured as well since neutrophils use ICAM-1 to adhere to the endothelium. (**A**) A representative immunoblot for MPO in the cortex of both groups. (**B**) MPO levels are increased in the ischemic hemisphere compared to the contralateral hemisphere in both groups (P < 0.001). SC-51089-treated rats showed significantly less MPO protein levels in the ischemic cortex compared to the vehicle-treated group (P < 0.05, unpaired two-tailed t-test). (**C**) A representative immunoblot for ICAM-1 in the cortex of both groups. (**D**) ICAM-1 levels are greatly increased in the ischemic hemisphere compared to the contralateral hemisphere in both groups (P < 0.001). ICAM-1 levels trended towards reduction in the ischemic cortex of SC-51089-treated rats compared to the vehicle (P = 0.16). Vehicle N = 10, SC-51089 N = 11 CXi = Cortex ipsilateral to stroke, CXc = Cortex contralateral to stroke.

Neutrophils are peripheral immune cells that are recruited to the site of injury and secrete MMPs as they migrate between endothelial cells to infiltrate into the tissue. Neutrophils are the predominant source of MMP-9 in ischemic stroke^33^. Myeloperoxidase (MPO) levels were used as an indicator of neutrophil infiltration into the brain parenchyma (Fig. 6A). MPO levels are dramatically increased in the ipsilateral cortex compared to the contralateral cortex in vehicle-treated rats (Fig. 6B, P < 0.001), and SC-51089 treatment decreased MPO levels in the ischemic cortex compared to the vehicle (Fig. 6B, *P* < 0.05). Reduced neutrophil infiltration may be due to reduced capacity for neutrophils to initiate migration. Intercellular Adhesion Molecule-1 (ICAM-1) is one of the adhesion molecules expressed by endothelial cells that allows neutrophils to adhere to the luminal surface of the endothelial cell wall and initiate migration. ICAM-1 levels were measured by immunoblotting (Fig. 6C). Dramatically increased ICAM-1 levels were observed in the ischemic hemispheres of both SC-51089- and vehicle-treated groups compared to the contralateral hemisphere (Fig. 6D, P < 0.0001). SC-51089 treated rats had reduced average ICAM-1 levels compared to the vehicle, although the difference between groups is not statistically significant (Fig. 6D, P = 0.16). Together, these data suggest that SC-51089 treatment may reduce neutrophil infiltration into the ischemic cortex, which is paralleled by reduced MMP-9/-3 levels and BBB disruption.

### SC-51089 protects TJPs against stroke-induced degradation

**Figure 7:**
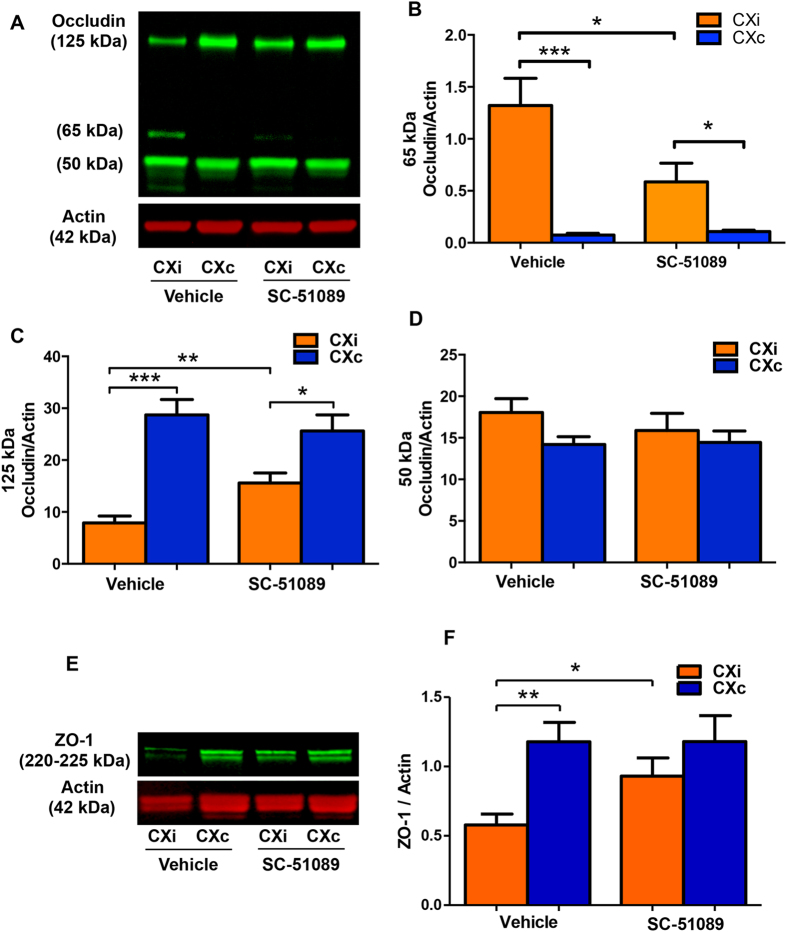
EP1 antagonist administration preserves tight-junction protein expression in the ischemic cortex. (**A**) An occludin band at approximately 65 kDa was observed in ischemic samples, but not in uninjured contralateral samples (presumably produced in response to injury). (**B**) Densitometric analysis of the 65 kDa isoform demonstrates increased levels in the ischemic hemisphere of both groups compared to the contralateral hemisphere (P < 0.001, P < 0.05, unpaired two-tailed t-test). Reduced levels of this protein were measured in the ischemic cortex of SC-51089-treated rats compared to the vehicle group (P < 0.05, unpaired two-tailed t-test). (**C**) An occludin band at approximately 125 kDa was observed in high amounts in the uninjured contralateral cortices and was reduced in ischemic samples. Densitometric analysis levels of the 125 kDa isoform demonstrates decreased levels in the ischemic hemisphere of both groups compared to the contralateral (P < 0.001, P < 0.05, unpaired two-tailed t-test) and preserved levels in the ischemic cortex of SC-51089-treated rats compared to the vehicle group (P < 0.01, unpaired two-tailed t-test). (**D**) Densitometric analysis of the 50-kDa occludin band showed no differences between groups. (**E**) Two ZO-1 bands were detected at 220-225kDa, which were degraded in ischemic samples. (**F**) ZO-1 is degraded in the ischemic hemisphere compared to the contralateral hemisphere in vehicle-treated rats (P < 0.01, unpaired two-tailed t-test). SC-51089 treatment preserved ZO-1 levels in the ischemic cortex compared to the vehicle (P < 0.05, unpaired two-tailed t-test) and differences between ischemic and contralateral hemisphere expression is not significantly different in SC-51089-treated rats (P = 0.29, unpaired two-tailed t-test). In panel **E**, actin appears as a double band due to non-reducing conditions during protein electrophoresis. Vehicle N = 10, SC-51089 N = 11. CXi = Cortex ipsilateral to stroke, CXc = Cortex contralateral to stroke.

We measured the protein levels of occludin and ZO-1, two proteins that are components of the BBB and substrates for MMPs. Occludin is a transmembrane protein that links adjacent endothelial cells and contributes to the paracellular barrier^4^. ZO-1 plays a role in the organization and regulation of the tight-junctions by interacting with occludin at the N-terminal and the actin cytoskeleton at the C-terminal^34^; these TJPs are degraded in focal ischemia^9^,^12^,^35^. The levels of occludin and ZO-1 were measured to elucidate whether SC-51089 reduces degradation of these TJPs in response to ischemia. We detected two occludin monomers (50 and 65 kDa), and one dimeric form (125 kDa) by immunoblotting (Fig. 7A). The 50-kDa occludin band is either a structural monomer or a degradation product^36^; there was no difference in the 50-kDa occludin band levels between groups (Fig. 7D, *P* = 0.43). The 65-kDa occludin band is likely a phosphorylated form produced in response to injury, as it is undetectable in the contralateral cortex and upregulated in the ischemic cortex, and found at a higher molecular weight; this band was decreased in SC-51089-treated animals compared to the vehicle (Fig. 7B, *P* < 0.05). The 125-kDa occludin isoform is possibly a structural dimer held together with irreducible disulfide bridges^36^; this band was protected from degradation in SC-51089-treated rats compared to the vehicle (Fig. 7C, *P* < 0.01). In both treatment groups, levels of ZO-1 were markedly reduced in the ipsilateral cortex compared to the contralateral cortex (Fig. 7E). ZO-1 was protected from degradation in SC-51089-treated rats compared to the vehicle (Fig. 7F, *P* < 0.05). Altogether, these data suggest that SC-51089 treatment reduces the disruption of the BBB by preserving occludin and ZO-1 levels, and likely reducing occludin phosphorylation.

### Effects of EP1 knockout on infarct size, blood-brain barrier permeability, and hemorrhagic transformation

**Figure 8:**
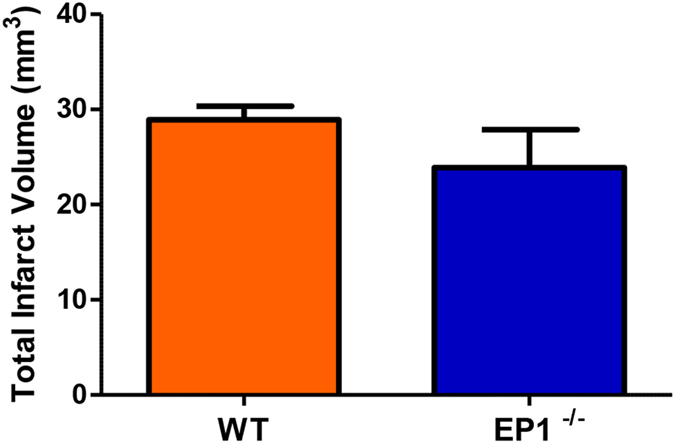
EP1 ^−/−^ mice trended towards smaller infarct volumes. Mice were subjected to 60 minutes of MCAO occlusion and euthanized 24 h after occlusion. Infarct volume was determined by 2,3,5-triphenyl-2H-tetrazolium chloride staining. Infarct volume is reduced in EP1^−/−^ mice (23.88 ± 4.008 mm^3^) compared to the wild-type (28.91 ± 1.428 mm^3^) although differences are not statistically significant (P = 0.26, unpaired two-tailed t-test). WT, N = 8; EP1^−/−^, N = 8. WT = wild-type, EP1^−/−^ = EP1 knockout.

We have demonstrated that pharmacological blockade of EP1 with SC-51089 decreases infarct volume and blood-brain barrier disruption in a rat stroke model. To confirm that our results were not due to off-target effects of SC-51089 administration, EP1^−/−^ mice were used to further ascertain the detrimental role of EP1 in stroke-induced BBB damage. EP1^−/−^ mice were subjected to 60 minutes of MCAO and euthanized at 24 h after reperfusion, similarly as performed before^26^. This endpoint was chosen since previous studies have shown increased MMP production and a dramatic BBB breakdown in the mouse brain at 24 h of reperfusion following transient MCAO^12^,^33^. Average infarct volume was reduced by 17% in EP1^−/−^ mice compared to the WT group, although the differences between groups were not statistically significant (Fig. 8, *P* = 0.26).

**Figure 9:**
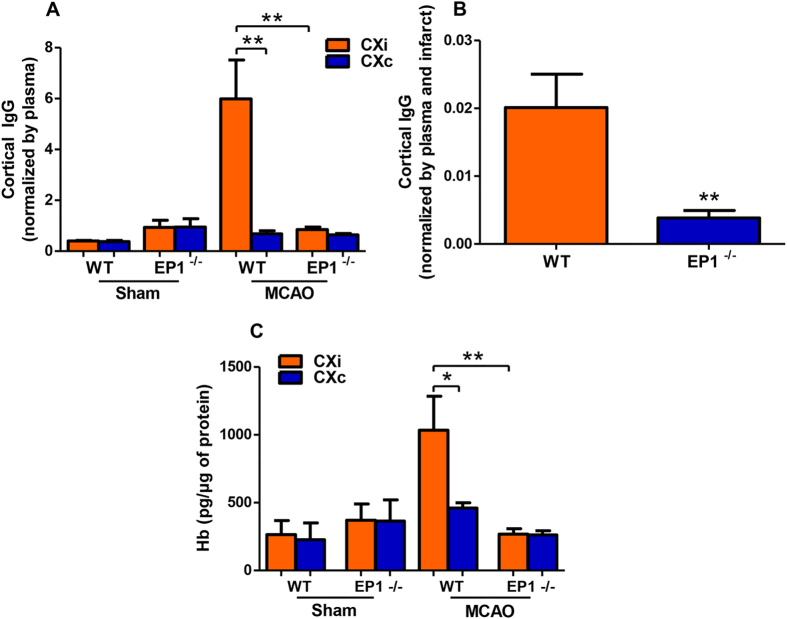
EP1^−/−^ mice show reduced blood-brain barrier permeability in the ischemic cortex. Cortical IgG levels were measured in sham-operated and ischemic animals, in WT and EP1^−/−^ groups, and normalized by plasma IgG levels and infarct volume. (**A**) Cortical IgG levels were divided by plasma IgG concentrations to yield a corrected measure of BBB permeability. IgG permeability is low in both sham-operated groups. IgG permeability is greatly elevated in the ischemic hemisphere of the WT MCAO group compared to the contralateral hemisphere (P < 0.01, unpaired two tailed t-test). IgG permeability is greatly reduced in the ischemic hemisphere of EP1^−/−^ MCAO mice compared to the WT (P < 0.01, unpaired two-tailed t-test). There are no significant differences between ischemic and contralateral hemispheres in EP1^−/−^ MCAO mice (P = 0.10, unpaired two-tailed t-test). (**B**) Cortical IgG levels normalized to plasma and infarct size show a dramatic reduction in EP1^−/−^ mice compared to WT controls (P < 0.01, unpaired two-tailed t-test). (**C**) Hemoglobin levels were used as a measure of the degree of hemorrhagic transformation. Hemoglobin levels are low in both sham-operated groups, and are significantly elevated in the ischemic hemisphere compared to the contralateral hemisphere in WT MCAO mice (P < 0.05, unpaired two-tailed t-test). Hemoglobin levels were reduced in the ischemic hemisphere of EP1^−/−^ MCAO mice compared to the wild-type (P < 0.01, unpaired two-tailed t-test). No significant differences were measured between ischemic and contralateral hemispheres of EP1 ^−/−^ MCAO mice (P = 0.90, unpaired two-tailed t-test). WT Sham, N = 3; EP1^−/−^ Sham, N = 3; WT MCAO, N = 8, EP1^−/−^ MCAO, N = 8. WT = wild-type; EP1^−/−^ = EP1 knockout, MCAO = middle cerebral artery occlusion.

Levels of IgG were measured in the cortices of EP1^−/−^ and WT mice as indicators of BBB permeability. Cortical IgG levels were divided by each animal’s corresponding plasma IgG concentration; IgG levels in the ipsilateral hemisphere were decreased in ischemic EP1^−/−^ mice compared to the ischemic WT (Fig. 9A, *P* < 0.01). The reduction in IgG concentrations in EP1^−/−^ mice persisted after normalizing by infarct volume (Fig. 9B, *P* < 0.01). We observed unusually high IgG levels in both hemispheres of the EP1^−/−^ sham-operated group and the contralateral hemisphere of the EP1^−/−^ MCAO group (Supplemental Fig. 2A). Analysis of IgG levels in the plasma of sham and MCAO groups revealed a significant interaction of genotype (accounting for ~37% of total variance, Supplemental Fig. 2B, P < 0.001), but not stroke (accounting for ~3% of total variance, P = 0.90) in accounting for the increases in plasma IgG concentrations observed in both EP1^−/−^ groups. In an additional control experiment, we measured IgG levels in plasma and cerebral cortex in both naïve EP1^−/−^ and WT mice. We found a highly significant increase in both plasma and cortical IgG levels in EP1^−/−^ compared to WT mice. Plasma IgG levels in naïve WT animals were 197.3 ± 15.1 μg/ml and values in EP1^−/−^ mice were 1988.4 ± 423.3 μg/ml (*P* = 0.0029, two-tailed unpaired t-test, n = 5 per genotype; mean ± SEM). A statistically significant increase in cortical IgG levels was also found in naïve EP1^−/−^ compared to WT. Ipsilateral IgG levels in the cortex of WT mice were 4.38 ± 0.14 pg/μg of protein *versus* 13.15 ± 1.14 pg/μg protein in EP1^−/−^ mice (*P* < 0.0001, two-tailed unpaired t-test, n = 5 per genotype; mean ± SEM). Since EP1 knockout increased baseline IgG levels in plasma, we utilized albumin levels in the brain as an additional marker of BBB permeability after stroke. EP1^−/−^ mice have dramatically reduced albumin levels in the ischemic cortex compared to the WT (Supplemental Fig. 2C, *P* < 0.001), an effect that persists after normalizing by infarct volume (Supplemental Fig. 2D, *P* < 0.01). No changes in albumin levels were found in the plasma of EP1^−/−^ compared to WT mice (Supplemental Fig. 2E).

To determine whether EP1 gene deletion reduces hemorrhagic transformation, hemoglobin levels were quantified in the cortices of WT and EP1^−/−^ mice in both sham and ischemic groups. Hemoglobin levels were decreased in the ischemic cortex of EP1^−/−^ mice compared to the WT (Fig. 9C, *P* < 0.01), indicating reduced hemorrhagic transformation.

### MMP-9 and MMP-3 activity is reduced in EP1^−/−^ mice

**Figure 10:**
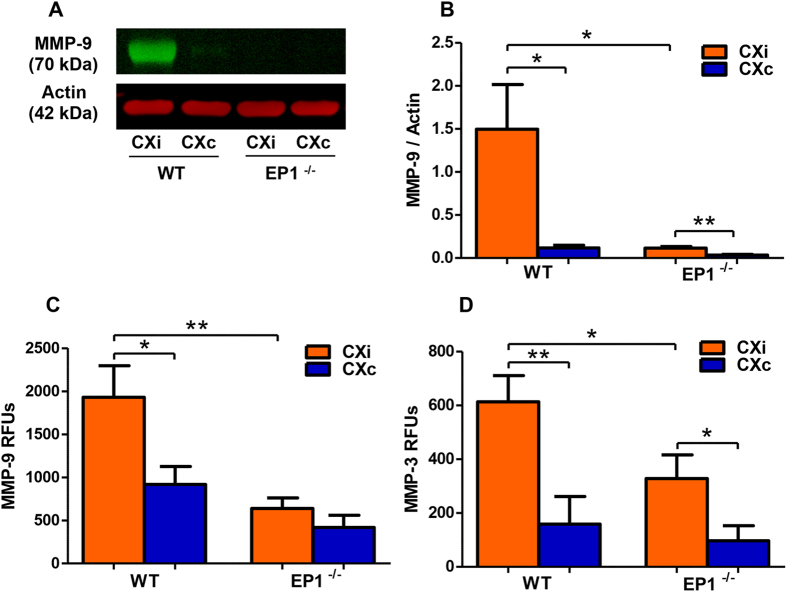
EP1^−/−^ mice show reduced MMP-9 protein levels and MMP-9/-3 activity. MMP-9/-3 protein levels were measured by immunoblotting, and enzymatic activity was quantified to determine if EP1^−/−^ mice show reductions consistent with the rat data. (**A**) A representative immunoblot of MMP-9 in the mouse cortex. (**B**) Densitometric analysis shows that MMP-9 is elevated in the ischemic hemisphere of both groups compared to the contralateral hemisphere (P < 0.05, P < 0.01 unpaired two-tailed t-test). EP1^−/−^ mice have dramatically reduced MMP-9 protein levels in the ischemic cortex compared to the wild-type (P < 0.05, unpaired two-tailed t-test). (**C**) A FRET peptide immunoassay was used to measure MMP-9 activity in the mouse cortex. MMP-9 activity was increased in the ischemic cortex of the WT group compared to the contralateral hemisphere (P < 0.05, unpaired two-tailed t-test), but not in the EP1^−/−^ group (P = 0.26, unpaired-two tailed t-test). MMP-9 activity was reduced in the ischemic cortex of EP1^−/−^ mice compared to the wild-type (P < 0.01, unpaired two-tailed t-test). (**D**) MMP-3 enzymatic activity was measured using a FRET peptide immunocapture assay. MMP-3 activity was increased in the ischemic cortex compared to the contralateral hemisphere in both groups (P < 0.01, P < 0.05, unpaired two-tailed t-test). MMP-3 activity was reduced in the ischemic cortex of EP1^−/−^ mice compared to the wild-type (P < 0.05, unpaired two-tailed t-test). WT, N = 8; EP1^−/−^, N = 8. CXi = Cortex ipsilateral to stroke, CXc = Cortex contralateral to stroke.

The levels of MMP-9 in the mouse cortices were quantified by immunoblotting (Fig. 10A). MMP-9 levels were elevated in the ischemic hemisphere of both groups compared to the contralateral hemisphere and were dramatically reduced in the ipsilateral cortex of EP1^−/−^ mice compared to the WT (Fig. 10B, *P* < 0.05). MMP-3 immunoblotting was attempted, but a viable signal could not be obtained. Endogenous MMP-9/-3 activity was quantified by a fluorometric immunocapture assay in the cortices of EP1^−/−^ and WT mice. Reductions in the activity of both MMP-9 (*P* < 0.01) and MMP-3 (*P* < 0.05) were observed in the ipsilateral cortices of EP1^−/−^ mice compared to the WT (Fig. 10C,D).

**Figure 11:**
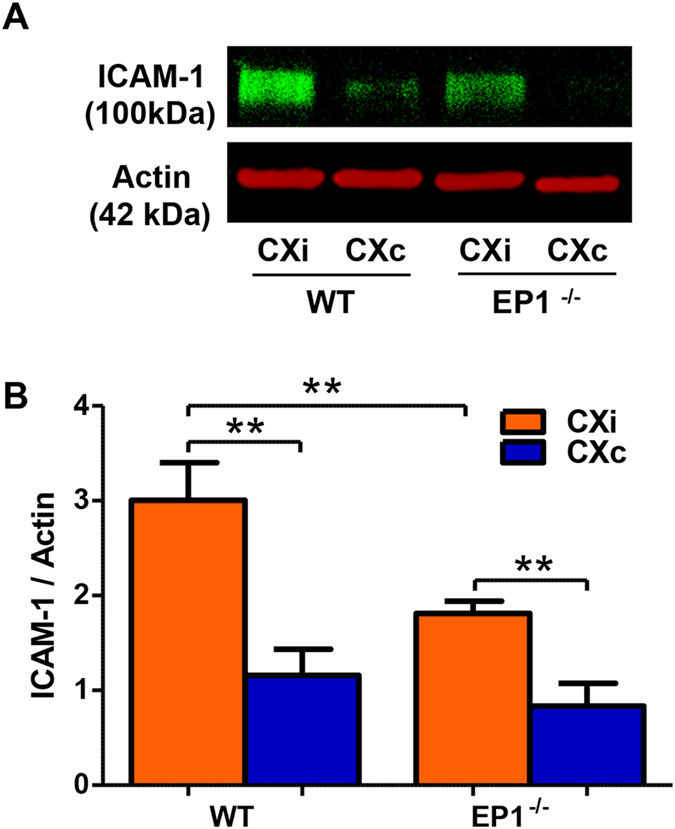
EP1^−/−^ mice show reduced ICAM-1 levels in the ischemic cortex. ICAM-1 levels were measured to determine whether EP1 deletion modulates ICAM-1 production in response to ischemia. (**A**) A representative Western blot for ICAM-1 in the mouse cortex. (**B**) Densitometric analysis of ICAM-1 levels showed an increase in the ischemic hemisphere compared to the contralateral hemisphere in both groups (P < 0.01, unpaired two-tailed t-test). ICAM-1 levels are significantly decreased in the ischemic EP1^−/−^ mice compared to the WT (P < 0.01, unpaired two-tailed t-test). WT, N = 6; EP1^−/−^, N = 8. CXi = Cortex ipsilateral to stroke, CXc = Cortex contralateral to stroke.

In order to corroborate our findings in the rat model, we attempted to measure MPO levels in the mouse brain by immunoblotting and ELISA, but we were unable to obtain a viable signal using either method. ICAM-1 levels were measured by immunoblotting (Fig. 11A,B); ICAM-1 levels in the ipsilateral hemisphere were elevated in both groups, but significantly reduced in the EP1^−/−^ group compared to the WT (P < 0.01, Fig. 11B).

## Discussion

In the present study, we demonstrated the neurovascular protective effect of EP1 inactivation in ischemic stroke. Post-ischemic treatment with SC-51089, an EP1 antagonist, or EP1 genetic deletion resulted in a significant decrease in protein levels and activity of MMP-9 and MMP-3 in the ischemic brain, and thereby reduced MMP-mediated neurovascular injury in an experimental transient ischemic stroke model in both rats and mice. That these BBB protective effects of EP1 inhibition are maintained across two different species is of significant translational relevance and increases the likelihood that this approach could also work in human stroke.

The EP1 antagonist used in this study, SC-51089, has been demonstrated to reduce infarct size and behavioral deficits in both transient^24^,^31^ and permanent^31^ models of ischemic stroke; with efficacy in both male and female mice and a wide window of therapeutic administration^31^. Our results are consistent with previous reports; post-ischemic injection of SC-51089 reduces total infarct volume 48 h post-MCAO in rats. We measured cortical and striatal infarct volumes separately, which revealed that the reductions in infarct volume were localized only to the cortex, with striatal infarct volume being almost identical between vehicle-and SC-51089-treated groups. These results suggest that pharmacological inhibition of EP1 likely reduces the progression of injury from the infarct core, mainly localized to the striatum, into the cortical penumbral region. Blood-brain barrier disruption progresses over a series of days and is a significant contributor to the progressive cell death occurring in the penumbra. Preserving the integrity of the neurovascular unit likely contributes to the reduction in cortical infarct volume observed in animals treated with an EP1 antagonist.

We observed a small but statistically insignificant reduction in total infarct volume at 24 h post-MCAO in EP1^−/−^ mice. However, previous reports have shown profound infarct volume reductions in EP1^−/−^ mice sacrificed at later time points: −35% at 72 h^24^ and −42% at 96 h^26^ compared to the wild-type, showing that the neuroprotective effect of EP1 deletion is seen at time points later than 24 h.

We are the first to show that *EP1* mRNA and protein levels are increased in the brain at 4 h and 48 h following ischemic stroke in rats. Pro-inflammatory signals such as TNFα have been shown to increase *EP1* mRNA in human amnionic WISH cells^37^. Thus, it is possible that the *EP1* regulation 4 h post-MCAO may be mediated by TNFα, although confirmation of this mechanism in the brain is outside the scope of this study. *EP1* mRNA increases at 48 h could be due to compensatory production by surviving resident brain cells. EP1 protein expression is increased 14 h after ischemia in the cortex, and then falls back to baseline levels. Increased expression of EP1 may result in increased susceptibility to PGE_2_-mediated neurotoxicity in the brain during the acute phase of ischemic injury.

IgG concentrations in the brain were measured to quantify BBB permeability in SC-51089-treated rats and EP1^−/−^ mice because IgG levels are extremely low in the uninjured contralateral cortex and greatly increased in the ischemic brain when the BBB is compromised. IgG levels in the ischemic cortices of SC-51089-treated rats were significantly lower compared to the vehicle, suggesting that the BBB could be more intact in the treated group. Since IgG is a soluble plasma protein, we divided the concentration of IgG measured in the cortex by the concentration of IgG in the plasma to account for variability between animals, and then by the stroke volume in the corresponding hemisphere to produce an approximate measurement of the amount of IgG per-unit of tissue volume. In SC-51089-treated rats, IgG levels were reduced in the ischemic hemisphere after normalization, which supports that the reductions in IgG levels in the rat brain are due to reduced neurovascular injury, and not merely due to a smaller infarct size in animals treated with the EP1 antagonist.

In EP1^−/−^ mice, the results are complicated by the fact that EP1 knockout increases circulating IgG levels several-fold. To our knowledge, this is the first report of an EP1-mediated mechanism modulating IgG production, demonstrating a previously unknown interaction between inflammation and immune system regulation. Cortical IgG levels were divided by the animal’s corresponding plasma IgG concentration, revealing significantly reduced IgG levels in the ischemic hemisphere of EP1^−/−^ mice. Another soluble plasma protein, albumin, was chosen as a second measure of BBB permeability due to the EP1 knockout/IgG interaction. Albumin levels were markedly reduced in the ischemic hemisphere of EP1^−/−^ mice, an effect that persists after normalizing by infarct volume. Hemorrhagic transformation is a serious consequence of ischemia that increases mortality in stroke patients, and occurs where BBB disruption is great enough to allow erythrocytes to pass into the parenchyma^32^. Hemoglobin levels were measured as an indicator of hemorrhagic transformation. Pharmacological blockade or genetic deletion of EP1 resulted in reduced hemoglobin levels in the ischemic brain. Collectively, these data indicate that inhibition of EP1 signaling reduces the degree of BBB disruption and hemorrhagic transformation following experimental cerebral ischemia.

MMP-9 and MMP-3 have been implicated in neurovascular injury and apoptotic cell death in ischemic stroke, in both humans and rodent models. In human ischemic stroke, perivascular neutrophils express MMP-9 and are associated with disrupted micro-vessels, hemorrhagic transformation, and basal lamina degradation^38^. In the peri-infarct region, microglia, macrophages, endothelial cells, and neurons express MMP-9^10^,^15^,^39^. In the clinical setting, MMP-9 levels in plasma have been correlated with fluid-attenuated inversion recovery hyperintensities^40^, hemorrhagic transformation^41^, blood-cerebrospinal fluid barrier disruption^42^, and poorer behavioral outcomes^43^. Extensive evidence in rodent models suggests that acute inhibition of MMP-9 and MMP-3 confers protection to the ischemic brain. In mice, MMP-9 knockout reduces fluid edema, infarct size, ZO-1 degradation, and BBB permeability^11^,^12^. Pharmacological inhibition of MMP-9 reduces infarct size, neurological deficits, DNA fragmentation, hemorrhagic transformation, and apoptotic cell death after ischemic stroke^44^,^45^,^46^. MMP-3 knockout limits BBB breakdown, reduces pro- and active MMP-9 expression in the brain, and reduces TJP degradation in models of neuroinflammation and cerebral ischemia^13^,^47^.

Findings from this study demonstrate that an EP1-mediated mechanism modulates MMP-9 and MMP-3 in the ischemic brain. Elucidating the mechanism by which EP1 inhibition reduces MMP levels in the ischemic brain is complicated since both resident brain cells and peripheral immune cells produce MMPs in response to ischemia^48^. It is possible that EP1 blockade reduces neuronal MMP-9 transcription. EP1 is expressed on neurons, and EP1 stimulation with PGE_2_ has been shown to phosphorylate NF-κB^49^. The MMP-9 promoter has an NF-κB binding domain^50^,^51^,^52^, thus inhibition of EP1 signaling may reduce transcription of MMP-9 in the brain. Although resident brain cells produce MMPs, infiltrating neutrophils have been demonstrated to be the major source of MMP-9 in cerebral ischemia and the extent of neutrophil infiltration correlates with stroke volume in humans^33^,^53^. Neutrophils are recruited to the brain following ischemia, where they adhere to the endothelial cell wall and migrate into the tissue. Neutrophils secrete MMPs and degrade TJPs as they pass through the neurovascular unit, increasing the permeability of the BBB^33^,^54^,^55^. If blockade of EP1 signaling inhibits the ability of neutrophils to adhere to the endothelial cell wall, this could explain decreased infarct volumes, decreased blood component extravasation, reduced MMP levels, preserved occludin, and ZO-1 levels in EP1 antagonist-treated rats and EP1^−/−^ mice. In the present study, MPO was used as an indicator of peripheral neutrophil infiltration. Significantly less MPO was measured in SC-51089-treated rats compared to the vehicle, suggesting reduced neutrophil infiltration in animals receiving the EP1 blocker. Previous research has shown that EP1 upregulates ICAM-1 levels in oral cancer cells^56^. ICAM-1 levels were measured to determine if EP1 modulates ICAM-1 in cerebral ischemia. ICAM-1 levels trended towards a decrease in the SC-51089-treated group, but EP1 gene deletion significantly reduced ICAM-1 expression in the ischemic cortex compared with WT mice. This may reflect a more profound effect of EP1 knockout compared to a pharmacological antagonist and/or differences between species in the mechanism(s) responsible for the increases in ICAM-1 levels in response to stroke. Nonetheless, decreased ICAM-1 in EP1^−/−^ mice suggests reduced neutrophil infiltration.

In addition to MMP-mediated BBB disruption our results show that SC-51089 treatment may reduce MMP-independent BBB permeability. A higher molecular weight occludin isoform was observed in ischemic samples but not in the unaffected contralateral hemispheres. This isoform is likely a phosphorylated and/or ubiquitinated occludin isoform. Cerebral ischemia phosphorylates occludin^57^, which attenuates interactions with ZO-1^58^. Treatment with SC-51089 significantly reduced the levels of this occludin isoform. A previous report utilizing an embolic rat model of ischemic stroke demonstrated that SC-51089 reduces tyrosine phosphorylation of occludin^59^. Others have demonstrated that ischemia-reperfusion injury can phosphorylate and ubiquitinate occludin^60^. Our data suggest that EP1 inhibition may also reduce BBB permeability by reducing post-translational modifications of occludin, consequently preserving the structural organization of the tight junctions and BBB functionality.

In conclusion, we have demonstrated that EP1 antagonism or *EP1* gene deletion limits BBB breakdown, attenuates hemorrhagic transformation, and significantly reduces MMP-9 and MMP-3 protein levels and activity following experimental ischemic stroke in rodents, which is likely due to reduced immune cell infiltration into the brain. These results suggest that inhibiting EP1 signaling may be a viable therapeutic strategy to reduce neurovascular injury in ischemic stroke. Despite the prominent role of MMP-9 and MMP-3 in BBB disruption in cerebral ischemia, therapeutic targeting of these proteases in humans is challenging because current pharmacological inhibitors produce a complete and unspecific inhibition of all members of the MMP family which results in unfavorable side effects and limits their clinical use^61^. Upstream inhibition of MMP production or activation may be an effective alternative to direct MMP inhibitors. Findings from our current study indicate that EP1 blockade could represent a novel alternative strategy to protect the BBB from MMP-9 and MMP-3-associated breakdown following ischemic stroke.

## Materials and Methods

### Animals

All procedures involving animals were performed in accordance with the approved guidelines of the National Institutes of Health (Bethesda, MD, USA) for the care and use of laboratory animals, the ARRIVE guidelines (https://www.nc3rs.org.uk/arrive-guidelines), and were approved by the Institutional Animal Care and Use Committee at the University of Florida (protocol approval number: 201106503). All efforts were made to minimize pain and distress to the animals. For the rat experiments, we purchased adult male Wistar rats (10-12 weeks of age; 280–320 g) from Harlan Laboratories (Indianapolis, IN, USA). All animals were acclimated to our animal facility for at least 7 days before any surgical procedure. Rats were housed in groups of two in a room with a controlled environment and a 12-h light/dark cycle. Animals had free access to food and water. For the experiments involving mice, we utilized adult male wild-type (WT) and EP1^−/−^ mice on the C57BL/6 background. Mice were bred and maintained in our animal facilities and were used when they were 8-12 weeks old (20–25 g). Mice had access to food pellet and water *ad libitum*, and were housed under reverse light cycle conditions.

### Surgical procedures to induce middle cerebral artery occlusion (MCAO)

Transient focal cerebral ischemia was induced by intraluminal insertion of a silicone-coated filament as described in detail in our previous reports^20^,^26^,^27^,^35^. Briefly, animals were anesthetized by isoflurane inhalation in medical-grade oxygen. Once surgical levels of anesthesia were attained, animals were placed on a water-circulating heat pad (Gaymar, TP-700, Torrington, CT, USA). The surgical area was shaved and swabbed with betadine followed by 70% ethanol. Surgery was performed using a stereomicroscope (Motic, SMZ-168, Richmond, BC, Canada). The anterior neck was opened with a midline vertical incision. Dissection medial to the right sternocleidomastoid muscle exposed the common carotid artery (CCA), external carotid artery (ECA), and internal carotid artery (ICA). The arteries were carefully separated from the vagus nerve and connective tissue. A nylon filament (4-0 for rats; 7-0 for mice), coated with silicone rubber, was advanced gently in the ICA to occlude the ostium of the middle cerebral artery (MCA) until a mild resistance was felt, approximately 18–20 mm from the carotid bifurcation in rats, and 9–11 mm in mice. In rats, MCAO was confirmed by a neurological exam performed after one hour of filament insertion. Only rats showing consistent contralateral circling and paw deficits were included in this study. In mice, successful MCAO was confirmed by a significant drop of more than 80% in cerebral blood flow from baseline as monitored by laser Doppler flowmetry (moorVMS-LDF Dual Channel Laser Doppler Blood Flow Monitor, Moor Instruments Inc., Wilmington, DE, USA). The occluding filament was kept in place for 90 min (rats) or 60 min (mice). At the end of the ischemic period, the animals were re-anesthetized and the filament was gently retracted to allow reperfusion of the ischemic region. The incision was closed with nylon sutures, povidone-iodine was applied, and animals were allowed to recover from anesthesia. Animals were injected subcutaneously with 0.05 mg/kg of buprenorphine hydrochloride (Buprenex) to minimize pain and allowed to fully recover in a warm cage (Thermocare ICS Warmer W-1 model 75W, Paso Robles, CA, USA).

### Drug Treatment

For the intravenous administration of vehicle or SC-51089, an EP1 receptor antagonist, a Micro-Renathane catheter was placed in the right distal segment of the femoral vein of ischemic rats at the time of MCAO surgery. SC-51089 (Cat. No. 10011561, Cayman Chemical, Ann Arbor, MI, USA) was freshly dissolved in 30% Kolliphor^®^ HS 15 (Cat. No. 42966, Sigma-Aldrich, Saint Louis, MO, USA) in physiological saline and given after 3 h of reperfusion (4.5 h after the onset of MCAO) at a dose of 3 mg/kg. Rats in the vehicle group were on the same treatment schedule and received the same volume of 30% Kolliphor^®^ HS15. Additional injections of SC-51089 or vehicle were administered after 18 and 28 h of reperfusion via the femoral vein catheter. Rats were randomly assigned to vehicle or treatment conditions.

### Infarct Volume Measurement

At the time of euthanasia, rodents were anesthetized with an overdose of sodium pentobarbital (150 mg/kg; i.p.) and perfused transcardially with ice-cold saline. The brains were removed, placed in a slicing matrix (Zivic Instruments, Pittsburgh, PA, USA) and cut into sections (six 2-mm sections in rat, seven 1-mm sections in the smaller mouse brain). The fourth section (starting rostrally) was frozen on dry ice for molecular analyses, while the rest of the sections were incubated in a 2% TTC solution at 25 °C for 30 minutes to stain for metabolically active tissue. TTC-stained sections were fixed in a 4% PFA solution in PBS, then placed rostral-side down on a HP Scanjet 8300 (Palo Alto, CA, USA), scanned at 600 dpi, and saved as a JPEG. Since the fourth section was removed for molecular analyses, the infarct size of the fourth slice was determined by scanning the caudal side of the third section.

Due to the swelling associated with edema in the ipsilateral hemisphere, measuring the area of infarcted tissue directly would overestimate the infarcted area. In this study, corrected infarct volume was calculated by measuring the area of TTC-stained cortical or striatal tissue on the contralateral side of the brain and subtracting the area of TTC-stained tissue on the ipsilateral side of the brain as described previously^62^. Adobe Photoshop CS5 was used to select and calculate areas in mm^2^ per brain slice. Infarct volume per slice was determined by multiplying the area of infarcted tissue by the thickness of the slice. Total infarct volume was calculated by summing individual slice infarct volumes.

### Brain tissue homogenization

Brain tissue homogenates were prepared from the fourth rostral section. Ipsilateral and contralateral hemispheres were separated, then cortical and striatal tissue was dissected out. The tissue samples were weighed and homogenized in a RIPA buffer containing 1% SDS, 1% sodium deoxycholate, 150 mM NaCl, 50 mM Tris-HCl pH 7.6, and 1% IGEPAL® CA-630 (Sigma-Aldrich) at 10 μL/mg of brain tissue. HALT Protease Inhibitor Cocktail, HALT Phosphatase Inhibitor Cocktail and 0.5 M EDTA (Cat. No. 78430; Cat. No. 78428; and Cat. No. 1860851, respectively; Thermo Fisher Scientific, Waltham, MA, USA) were added at 10 μL/mL of homogenization buffer immediately before use. Brain tissue was homogenized with a Tissue-Tearor homogenizer (BioSpec Inc., Bartlesville, OK, USA) then sonicated twice using a Vibra-Cell™ sonicator (Sonics & Materials Inc., Newtown, CT, USA) with 15 minutes of equilibration on ice between sonications. Resulting tissue homogenates were centrifuged at 14,000× g for 20 min at 4 °C in an Eppendorf Microcentrifuge Model 5430R (Hamburg, Germany), and the supernatants were aliquoted and stored at −80 °C until use.

### Western Blotting

Forty or fifty μg of protein (for mouse and rat, respectively) were reduced in 5% β-mercaptoethanol (Sigma-Aldrich) at 100 °C for 10 min and loaded into 4–20% Mini-PROTEAN TGX gels (Bio-Rad, Hercules, CA, USA). Proteins were separated at 200V for 45 min in a 0.1% SDS Tris-glycine running buffer and the gels were subsequently incubated for 10 min in a Tris-glycine buffer containing 10% methanol. Proteins were transferred to Immobilon-FL PVDF membranes (Millipore, Billerica, MA, USA) using a Trans-Blot Turbo (Bio-Rad) semi-dry transfer system. The membranes were blocked in 5% non-fat milk in TBS for 1 h at 25 °C. Primary antibodies against proteins of interest: rabbit polyclonal anti-EP1 Cat. No. 101740, 1:2,000, Cayman Chemical; rabbit monoclonal anti-MMP-9 (rat) Cat. No. ab76003, 1:5,000, Abcam (Cambridge, MA, USA); rabbit polyclonal anti-MMP-9 (mouse) Cat. No. SC-6841-R, 1:500, Santa Cruz Biotechnology (Dallas, TX, USA); rabbit polyclonal anti-MMP-3 (rat and mouse) Cat. No. ab53015, 1:1,000, Abcam; rabbit monoclonal anti-occludin Cat. No. ab167161, 1:1,000, Abcam; rabbit polyclonal anti-ZO-1 Cat. No. 61-7300, 1:1,000, Life Technologies (Carlsbad, CA, USA); rabbit polyclonal anti-MPO Cat. No. SC-16128-R, 1:700, Santa Cruz Biotechnology; goat polyclonal anti-ICAM-1 (rat and mouse) Cat. No. AF583, 1:800, R&D Systems (Minneapolis, MN, USA) were added to 5% milk in TBST and incubated overnight at 4 °C. The membranes were washed four times in TBST and probed with IRDye 800CW goat anti-rabbit or donkey anti-goat secondary antibodies (1:30,000; Li-Cor, Lincoln, NE, USA) in 5% milk in TBST w/0.01% SDS for 1 h at 25 °C. The membranes were scanned on an Odyssey infrared scanner (Li-Cor) and band intensity was measured using Image Studio V2.0. The membranes were probed with anti-β-actin antibody (A1978, 1:10,000, Sigma-Aldrich) for 1 h at 25 °C followed by incubation with IRDye 680LT donkey anti-mouse (1:40,000; Li-Cor) for 1 h at 25 °C. The membranes were scanned and the signal of the protein of interest was divided by the actin signal to correct for potential loading and transfer variations between lanes/gels.

### ELISAs

Various proteins normally found in the blood, but not found in the healthy brain, were used as indicators of BBB permeability. Immunoglobulin G (IgG), hemoglobin (Hb), and albumin concentrations were measured. ELISA kits were purchased from Immunology Consultants Laboratory, Inc. (Portland, OR, USA) and used according to the provided instructions (rat IgG, Cat. No. E-25G; rat Hb, Cat. No. E-25HM; mouse IgG, Cat. No. E-90G; mouse Hb, Cat. No. E-90HM; mouse albumin, Cat. No. E-90AL). Absorbance was measured at 450 nm using a Synergy HT Multi-mode plate reader (BioTek, Winooski, VT) running Gen5™ data analysis software. IgG concentrations were measured using 50 μg of total protein, Hb concentrations using 10 μg of total protein, and albumin concentrations using 3 μg of total protein. Plasma was diluted 1:20,000 for IgG quantification and 1:500,000 for albumin quantification. Corrected values were obtained by dividing measured protein concentrations by the infarct volume of the fourth brain section.

### Fluorometric immunocapture assay of MMP enzymatic activity

Enzymatic activity of MMP-9 and MMP-3 was measured using a fluorescence resonance energy transfer (FRET) peptide immunoassay as previously described by our group^63^,^64^. High-binding 96-well plates (Greiner Bio-One, Monroe, NC, USA) were coated with 200 μg/mL of protein A/G (Cat. No. 1003-01, ScienCell, Carlsbad, CA, USA) in a carbonate/bicarbonate buffer at a pH of 9.4 for 2 h at 25 °C. The wells were aspirated and washed three times with 200 μL of TCNB (50 mM Tris, 10 mM CaCl_2_, 150 mM NaCl, 0.05% Brij® L23). Polyclonal rabbit anti-MMP-9 or anti-MMP-3 antibodies (Cat. No. SC-6841-R; Cat. No. SC-6839-R, Santa Cruz Biotechnology) were added to the wells at 0.5 μg/well and bound for 2 h. The wells were aspirated and washed three times before adding samples (50 μg of total protein) in duplicate into the wells containing the protein A/G-antibody complex and allowed to bind overnight at 4 °C. The wells were aspirated, washed, and 5-FAM/QXL™ 520 FRET peptides (Substrate III Cat. No. AS-60570-01 or Substrate XIII Cat. No. AS-60580-01, Anaspec (Fremont, CA, USA), for MMP-9 or MMP-3, respectively) were added to the wells. The plates were incubated at 37 °C and read at excitation/emission wavelengths of 485/528 nm after 24 and 48 h of incubation in a Synergy HT Multi-mode microplate fluorescence reader running Gen5™ data analysis software (BioTek). Baseline fluorescence was determined from the average of two substrate control wells, which was subtracted from the average of each pair of sample wells to give a final relative fluorescent unit (RFU) value.

### qRT-PCR

*EP1* mRNA expression in response to ischemia was measured using qRT-PCR in sham-operated, 4 h, 14 h, 24 h, and 48 h post-MCAO rats. Following perfusion with ice-cold saline, the brains were placed in a slicing matrix (Zivic Instruments) and sectioned into six 2-mm sections. The third section was separated into ipsilateral and contralateral hemispheres, and cortical tissue was separated from striatal tissue. Tissue samples were placed in RNAlater RNA Stabilization Reagent (Cat. No. 76106, Qiagen, Germany) at 10 μL/mg of tissue, kept at 4 °C for 24 h and then placed at −20 °C until further processing. Using the Aurum Total RNA Fatty and Fibrous Tissue Kit (Cat No. 732-6830, Bio-Rad) the RNA was extracted from the tissue according to the provided instructions. Total cDNA was generated in a T100 thermal cycler (Bio-Rad) using iScript Supermix (Cat. No. 170-8841, Bio-Rad). Quantitative real-time PCR was performed using the following primers: *EP1* forward, 5′-CCT GCT GGT ATT GGT GGT GTT-3′, *EP1* reverse, 5′-CAG GAT CTG GTT CCA CGA CG-3′; *Ywhaz* forward, 5′-TGT TCT AGC CTG TTT CCC CG-3′, *Ywhaz* reverse, 5′-ACG ATG ACG TCA AAC GCT TC-3′. *Ywhaz* was used for normalization since gene expression does not significantly change in response to ischemia^65^, which was confirmed in our preliminary pilot study. The samples were loaded in quadruplicates with 5 μL of SsoAdvanced Universal SYBR Green Supermix (Bio-Rad), 1 μL of forward and reverse primers (IDT, Coralville, IA, USA), 1 μL of nuclease-free water, and 20 ng of cDNA per well. The plate was spun down and the PCR reaction proceeded under the following conditions: 95 °C 30 s, (95 °C 5 s, 60 °C 30 s) for 40 cycles, followed by a melt curve calculation (start - 65 °C 5 s, +0.5 °C/cycle for 60 cycles) in a CFX96 C1000 Touch thermal cycler running CFX Manager 3.1 (Bio-Rad). Cq values for each replicate well were averaged to give a final Cq value for each animal. Relative normalized expression was determined by a ΔΔCq calculation where Cq values were normalized to *Ywhaz* expression to give ΔCq values, which were normalized to the expression in the ipsilateral cortex of the sham group to produce the final ΔΔCq value.

### Immunohistochemistry

Rodents were perfused with PBS followed by 4% PFA. The brains were removed and placed in a 4% PFA solution and kept at 4 °C overnight and then transferred to 30% sucrose in PBS for 48 h. The brains were frozen in Tissue-Tek^**®**^ optimum cutting temperature (O.C.T.) embedding medium, sectioned at 40 μm in a cryostat, placed in a cryoprotectant solution (30% ethylene glycol, 30% glycerol, 10% PBS, 30% water; adjusted to pH 7.4), and kept at –20 °C until use. Free-floating sections were washed four times with TBS w/ 0.1% Triton X-100, and blocked with 5% normal goat serum and 1% bovine serum albumin for 1 h at 25 °C. Antibody incubations were performed in 12-well plates containing 2 mL of TBS w/ 0.1% Triton X-100 containing 0.5% BSA. Sections were incubated for two nights at 4 °C on an orbital shaker at 50 rpm with primary antibodies against proteins of interest: rabbit polyclonal anti-EP1 Cat. No. ab93479, 1:250, Abcam; mouse monoclonal anti-NeuN Cat. No. MCA-1B7, 1:1,000, EnCor Biotechnology (Gainesville, FL, USA); mouse monoclonal anti-RECA-1 Cat. No. MCA970GA, 1:1,000, AbD Serotec (Raleigh, NC, USA); mouse monoclonal anti-GFAP Cat. No. MCA-5C10, 1:1,000, EnCor Biotechnology; mouse monoclonal anti-CD11b Cat. No. MCA275GA, 1:1,000, AbD Serotec. Sections were washed three times and secondary antibodies (anti-rabbit Alexa Fluor 488 Cat. No. 111-545-144, 1:1,000, Jackson IR (West Grove, PA, USA); anti-mouse Cy3 Cat. No. 115-165-166, 1:1000, Jackson IR) were added to 0.5% BSA in TBS and incubated for 2 h at 25 °C. Sections were washed three times and incubated in 100 nM DAPI for 10 s, dipped in water, mounted with Fluoromount (Cat. No. F4680, Sigma-Aldrich), and stored at 4 °C until imaging. Images were obtained on an Olympus disc scanning unit (DSU) motorized IX81 spinning disc confocal microscope at 60× magnification using a water immersion lens and DAPI, FITC, and TRITC filters for the blue, green, and red channel acquisition, respectively.

### Statistical analyses

Data are shown as the mean ± SEM. GraphPad Prism 5 was used to conduct statistical tests. A two-tailed unpaired Student’s t-test, one-way ANOVA followed by a Tukey post-test, or two-way ANOVA followed by a Bonferroni post-test was used. A *P*-value of less than 0.05 was considered statistically significant.

## Additional Information

**How to cite this article**: Frankowski, J. C. *et al*. Detrimental role of the EP1 prostanoid receptor in blood-brain barrier damage following experimental ischemic stroke. *Sci. Rep*. **5**, 17956; doi: 10.1038/srep17956 (2015).

## Supplementary Material

Supplementary Information
