# Supplementary material for: Detrimental role of the EP1 prostanoid receptor in blood-brain barrier damage following experimental ischemic stroke
Source: Sci Rep. 2015 Dec 9;5:17956. doi: 10.1038/srep17956 (PMC4673693; doi:10.1038/srep17956)
Supplement: Supplementary Information [file srep17956-s1.pdf]

SUPPLEMENTARY INFORMATION

Detrimental role of the EP1 prostanoid receptor in blood-brain barrier damage following experimental ischemic stroke

Jan C. Frankowski[1], Kelly M. DeMars[1], Abdullah S. Ahmad[2], Kimberly E. Hawkins[1], Changjun Yang[1], Jenna L. Leclerc[2], Sylvain Doré[1,2], Eduardo Candelario-Jalil[1,*]

[1]Department of Neuroscience, McKnight Brain Institute, University of Florida, Gainesville, FL 32610, USA

[2]Department of Anesthesiology, University of Florida, Gainesville, FL 32610, USA

Address correspondence to:

Eduardo Candelario-Jalil, Ph.D.

Department of Neuroscience, McKnight Brain Institute, 1149 SW Newell Drive, University of Florida, Gainesville, FL 32610. Phone: (352)-273-7116. Fax: (352)-392-8347. ecandelario@ufl.edu

This file includes 2 supplementary figures

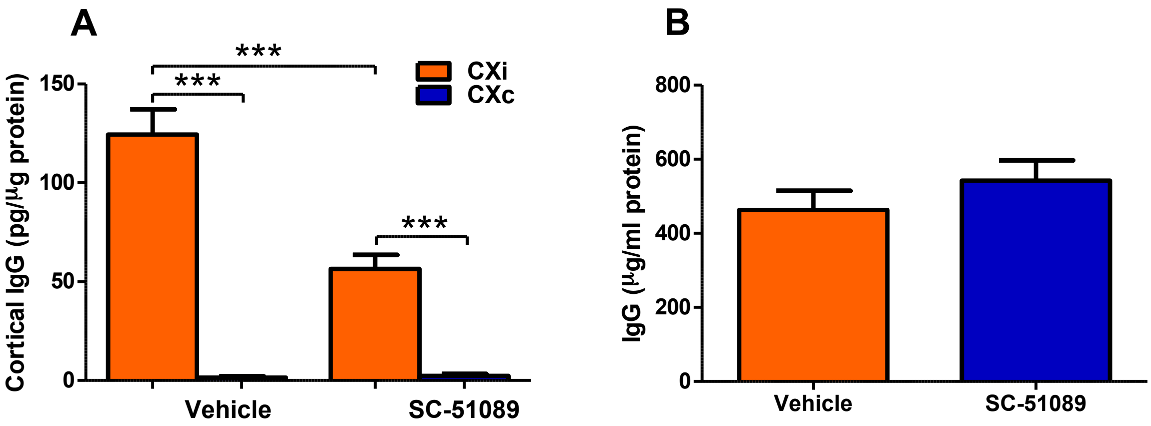

Supplemental Figure 1: IgG levels in the rat cortex and plasma

Immunoglobulin G was used as a measure of blood-brain barrier permeability since it is a soluble plasma protein that is not found in significant amounts in the healthy brain. IgG levels in plasma were measured to determine if treatment affects circulating IgG levels and to normalize cortical IgG levels by the plasma IgG levels. **A:** IgG levels were measured in the cortices of vehicle- and SC-51089 treated rats. IgG levels were greatly increased in the ischemic hemisphere of both groups compared to the contralateral hemisphere ($P<0.001$, unpaired two-tailed t-test). IgG levels were reduced in the ischemic cortex of SC-51089 treated rats (60.8 ± 6.8 pg/µg N=11) compared to the vehicle-treated group (140.7 ± 9.9 pg/µg) ($P<0.0001$, two-tailed unpaired t-test). **B:** IgG levels in the plasma are not significantly different in SC-51089-treated rats compared to the vehicle ($P=0.31$, unpaired two-tailed t-test). Vehicle N=10, SC-51089 N=11

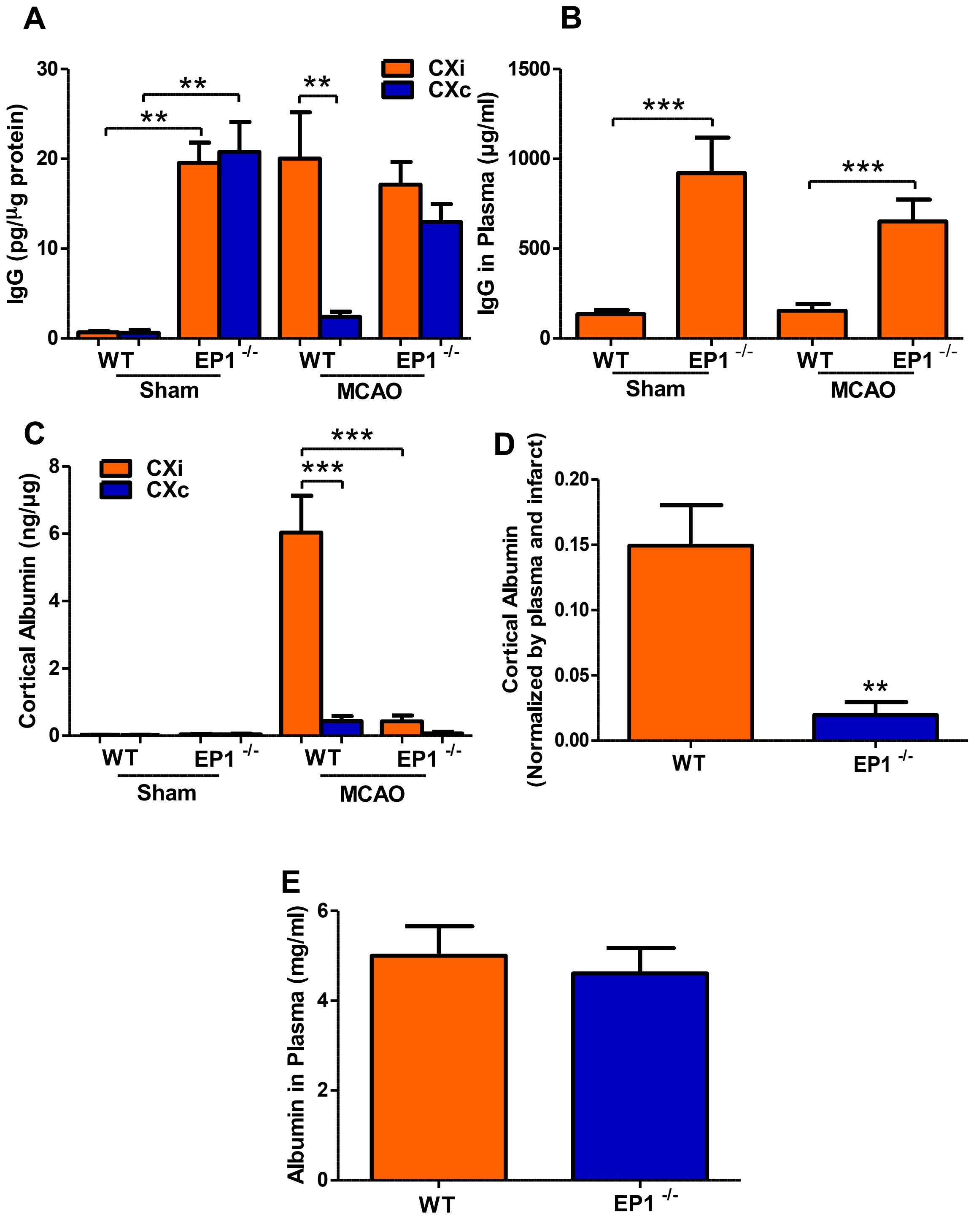

Supplemental Figure 2: EP1 deletion increased IgG levels in the brain and plasma, and significantly reduced albumin extravasation into the ischemic cortex

A: IgG levels were measured in the cortices of sham-operated and MCAO-operated mice, in both EP1$^{-/-}$ and wild-type groups. The sham group did not undergo stroke surgery and IgG levels are very low in both hemispheres in wild-type mice, although IgG levels are greatly elevated in

both hemispheres of $EP1^{-/-}$ sham-operated mice compared to the wild-type (P<0.01, P<0.01, unpaired two-tailed t-tests). IgG levels are not significantly different in the ischemic hemisphere between $EP1^{-/-}$ and wild-type mice subjected to MCAO (P=0.62, unpaired two-tailed t-test). IgG levels are not significantly different between the ipsilateral and contralateral hemispheres of $EP1^{-/-}$ subjected to MCAO (P=0.22, unpaired two-tailed t-test). **B:** Measurement of IgG levels in the plasma of all mouse groups revealed that EP1 knockout, but not MCAO, significantly increased circulating IgG levels (Genotype: 37% of total variation P<0.001, Stroke: 3% of total variation P=0.27, Interaction: 0.04% of total variation P=0.90, two-way ANOVA) IgG levels are 2.9-fold higher in the $EP1^{-/-}$ sham group and 6.3-fold higher in the $EP1^{-/-}$ MCAO group compared to the wild-type. **C:** Cortical albumin levels were measured as an additional measure of BBB permeability. Cortical albumin levels are greatly increased in the ischemic hemisphere of the WT group compared to the contralateral hemisphere (P<0.001, unpaired two-tailed t-test) Albumin levels were reduced in the ischemic cortices of $EP1^{-/-}$ mice compared to the wild-type (P<0.001, unpaired two-tailed t-test). **D:** Cortical albumin levels were normalized by infarct volume, normalized data shows that $EP1^{-/-}$ mice have reduced albumin per unit of infarcted tissue compared to the wild-type (P<0.01, unpaired two-tailed t-test). **E:** Levels of albumin in plasma were not different between WT and $EP1^{-/-}$ mice. CXi = Cortex ipsilateral to stroke; CXc = Cortex contralateral to stroke WT Sham N=3, $EP1^{-/-}$ Sham N=3, WT MCAO N=8, $EP1^{-/-}$ MCAO N=8. WT = wild-type; $EP1^{-/-}$ = EP1 knockout, MCAO = middle cerebral artery occlusion
